# Why do we Need to Control Alcohol Use Through Legislative Measures? A South East Asia Perspective?

**DOI:** 10.4103/0970-0218.62583

**Published:** 2010-01

**Authors:** Sahoo Saddichha, Narayana Manjunatha, Christoday Raja Jayant Khess

**Affiliations:** Department of Psychiatry, National Institute of Mental Health and Neurosciences (NIMHANS), Bangalore, India; 1 Department of Central Institute of Psychiatry, Ranchi, India

**Keywords:** Alcohol, legislation, opioids, policy, public health

## Abstract

**Background:**

Even though prevalence of alcohol use in the world is very high, it has not been brought under legal control in several countries, contrary to other controlled substances like opium, cocaine, cannabis, and so on.

**Aim:**

To demonstrate the similarities in both alcohol and opioid dependence by comparing and contrasting the course of clinical dependence for both substances.

**Patients and Methods:**

Consecutively admitted patients during the period August 2005 to May 2006, in the Center for Addiction Psychiatry, Central Institute of Psychiatry, Ranchi, India, with ICD-10 (DCR) diagnosis of alcohol dependence syndrome or opioid dependence syndrome were recruited for the study and administered the alcohol or other drug (opioid) section of SSAGA-II, respectively, and the data was entered in the corresponding tally sheet.

**Results:**

The total sample size was 150, of which 112 consented to participate. Eighty-one (72%) were alcohol-dependent and 31 (28%) were opioid-dependent. Mean ages of the patients of alcohol dependence for opioid dependence was 35.16 ± 10.2 compared to 26.09 ± 5.65. Mean age of onset of alcohol and opioid use were similar (18.72 ± 6.84 and 20.73 ± 3.93 years, respectively). Patterns of dependence were also similar for both substances, from the first criteria to dependence (0.49 years for alcohol versus 0.64 years for opioids), and from the appearance of the second criteria to dependence (0.24 years versus 0.28 years).

**Conclusion:**

This study recommends alcohol to be treated on par with opioids and calls for legislations for the control of alcohol, uniformly, across the world, as a public health policy, on the lines of the Framework Convention for Tobacco Control.

## Introduction

“Safe drinking is a delusion and a smokescreen to promote alcohol”

—Mohan D

Alcohol has been used in human societies at least since the beginning of recorded history, and throughout this time, humans have also been arguing about its merits and demerits. The debate still simmers today, with a lively back-and-forth over whether alcohol is good or bad for you. The consensus is that alcohol is both a tonic and a poison. Although moderate drinking seems to be good for the heart and circulatory system, and probably protects against type 2 diabetes and gallstones, heavy drinking is, unfortunately, a major cause of preventable death in most countries. Alcohol affects the body in many different ways. It affects not just the drinkers themselves, but may touch their families, friends, and communities, often involving violence and accidents.(1) It has been implicated in 40% of violent crimes,(2) 15% of drowning,(3) and is the cause of one in seven road traffic deaths.(4) Yet, the use of alcohol only seems to be increasing. According to estimates made by the World Health Report,(5) at least 10 thousand million people throughout the world regularly use alcohol. India has also been deeply affected where the intake of alcohol has so permeated into the culture that it is no longer acknowledged as a drug or even as a problem.(6)

India has the unique distinction of having some of the most varied varieties of alcoholic beverages. Country liquor, from locally available cheap raw material, such as, sugarcane, rice, palm, coconut, and cheap grains is available as arrack, desi sharab, tari, and toddy. Home-distilled alcohols are also popular such as *handia, chhun, apong, Zu, Rohi,* and *mahua.* It is no wonder that even with one in three people in India falling below the poverty line, alcohol use continues to be rampant causing adverse economic effects. These include reduced wages (because of missed work and lowered efficiency on the job, increased medical expenses for illness and accidents, legal cost of drink-related offences, and decreased eligibility of loans).(7) Industry association sources estimate that 15 to 20% of absenteeism and 40% of accidents at work are due to alcohol.(8) Alcohol use among industrial workers is increasing and this has led to an increase in alcohol-related sickness and absenteeism. The annual loss due to alcohol-related problems in work places is between Rs. 70,000 to 80,000 million.(9) A study looking at the prevalence and association of hazardous drinking in a male industrial worker population, in India, found that hazardous drinking was significantly associated with severe health problems, such as head injuries and hospitalizations.(10) In addition, alcohol use has been linked to 15 to 20% of traumatic brain injuries,(11) 20% to cases of domestic violence,(9) and accounts for over a fifth of all hospital admissions.(12) In India while gains in terms of revenue from alcohol sales were INR 216 billion every year, losses from the adverse effects of alcohol were estimated to be INR 244 billion, apart from the other immeasurable effects due to alcohol consumption. Therefore, the gain from the revenue earned from excise taxes ends up being spent to counter the effects of alcohol use in the medium and long-term.(13)

However with increasing globalization, there has also been increased acceptance and use of alcohol, which has now achieved serious ramifications. There is, therefore, an urgent need for reduction in the demand of alcohol, both legal and illegal, which may otherwise lead to numerous health, family, and societal consequences. Similar to the Narcotic Drugs and Psychotropic Substances (NDPS) Act, 1985,(14) in India, which provides the current framework for drug abuse control and sale in this country, there need to be similar provisions for the distribution and use of alcohol. However, legal control is yet lacking with a lack of consensus among clinicians on the harms and rates of dependence. There is a perception that the rates of conversion and the clinical course of alcohol dependence are different when compared to other legally controlled drugs like opium. The last reason is what can be corrected through systematic clinical studies, which till date have not been carried out. We therefore aimed at demonstrating the similarities in both alcohol and opioid dependence, by comparing and contrasting the course of dependence for both substances. We hypothesized that there would be no difference in the clinical course of dependence, which would follow a similar pattern across the lifeline, for both substances, hence making it imperative that both substances be treated equally.

## Patients and Methods

Patients, admitted consecutively, during the period October 2005 to August 2006, to the Center for Addiction Psychiatry (CAP), Central Institute of Psychiatry (CIP), Ranchi, India, with diagnosis of ICD-10 DCR(15) alcohol dependence syndrome (ADS)/Opioid dependence syndrome (ODS), and who gave written informed consent, were recruited for this study. The study protocol was approved by the Institute's ‘Ethics Committee’. The study was conducted at the CAP, CIP, Ranchi, India, which is a premier institute, in Eastern India, working under the aegis of the Ministry of Health and Family Welfare, Government of India, for postgraduate training in mental health, and has a large clinical service capacity of total 673 psychiatric inpatient beds, including a separate 30 bedded CAP. CAP treats over 500 persons/year including more than 350 alcohol-dependent inpatients per year, with nearly 100% bed occupancy. It has a wide catchment area and serves as a primary center for the people living in the immediate vicinity and a tertiary referral center for nearby states of India as well as neighboring South Asian countries like Nepal, Bhutan, and Bangladesh. Subjects who met criteria for other substance dependence, those who had other comorbid psychiatric disorders or general medical conditions requiring additional treatment, and patients who had cognitive impairment with Mini-Mental Status Examination,(16) with screening scores of less than 24, were excluded from the study.

Patients who fulfilled the study criteria and gave written informed consent were interviewed using the alcohol section of the Semi-Structured Assessment for the Genetics of Alcoholism-II(17) (SSAGA-II), after detoxification. SSAGA-II is a polydiagnostic instrument developed by the Collaborative Study on the Genetics of Alcoholism, team designed to assess the physical, psychological, and social manifestations of alcohol and other psychoactive substances, as well as, other psychiatric disorders according to ICD-10 and DSM-IV. It also has items related to each criteria of dependence of ICD-10 DCR. The reliability and validity of SSAGA has been established,(17,18) and it has been used widely in many international studies.(19–24) It also provides the diagnosis of major psychiatric disorders in DSM IV and provides a complete diagnosis according to DSM III R, DSM IV, and ICD 10. The alcohol section of SSAGA II contains 45 multipart items. SSAGA has good concordance with the schedule for Clinical Assessment in Neuropsychiatry(25) (SCAN) and has good Kappa value (K = .63) for alcohol and opioid dependence.(18) SSAGA-II permits a detailed evaluation of the first onset of each criteria of dependence according to ICD 10. ICD 10 uses the presence of three or more of the following criteria to diagnose dependence: tolerance, craving, salience or withdrawal, in the absence of drug, loss of control, and persisting use despite clear evidence of harmful consequences.

Relevant information of patients was also corroborated from their respective case record file (CRF) completed at the time of admission. In case of discrepancy of any items, it was discussed with the patient to come to a consensus. As it was a retrospective recall study, questions were framed individually to trigger the recall, using anchor questions to personal and impersonal or important social events and defining the technical terms.(26) At the end of the interview, data was transferred to the ICD 10 tally sheet of the respective items in the alcohol section of SSAGA II. Among the first age/s of appearance of items of each criterion, we considered the earliest age of appearance of any item, as the age of the first appearance of the respective criteria of dependence (ICD-10 DCR). We considered the age of development of ICD-10 dependence syndrome as the age of onset of the third consecutive criterion, with the simultaneous presence of the other two criteria (among the six criteria of ICD-10 DCR). Statistical analysis was done by performing the T-test for continuous variables and Chi-square for categorical variables.

## Results

The number of approached patients were 150, out of which 115 (76%) agreed to the SSAGA interview. There were no differences between the people who consented and those who refused. A further three questionnaires were rejected as patients left midway in the study. Therefore, of a total sample size of 112, 81(72%) were found to be alcohol-dependent and 31 (28%) were opioid-dependent. The mean age of the alcohol users was 35.16 ± 10.2 compared to the opioid users, whose mean age was 26.09 ± 5.65, and this difference was found to be, statistically, highly significant (*P* < 0.001). There were no differences among residence, occupation, and education (summarized in Table 1).

**Table 1:** Sociodemographic characteristics of study samples

Characteristics	Alcohol users group (N = 81)	%	Opioid users group (N = 31)	%	χ^2^/t-test	df	*P* value
Age	35.16 ± 10.2		26.09 ± 5.65		4.670	110	<0.001**
Residence					1.442	110	0.152
Rural	20	24.7	3	9.7			
Urban	61	75.3	28	90.3			
Education	11.69 ± 3.98		10.51 ± 3.49		3.230	1	0.072
Occupation					8.293	4	0.081
Professional and semi-pro	22	27.2	3	9.7			
Skilled and semi-skilled	40	49.4	20	64.5			
Unemployed	7	8.6	2	6.5			
Retired	5	6.2	0	0			
Students	7	8.6	6	19.4			

T-test has been used for continuous variables and Chi-square for categorical variables

*significance at *P*<0.05

** Significance at *P*<0.001

The mean age at onset of alcohol use in this study was 18.72 years (SD-6.84) compared to opioid use being initiated at 20.73 years (SD-3.93). Age at onset of ICD-10 (DCR) dependence was 27.51 years (SD-9.28) in alcohol users compared to it being 22.05 (SD-3.98) years in opioid users. The duration from onset to dependence also differed significantly between both groups.

As the ages at presentation differed significantly between both groups, we calculated the ratio of durations from the first and second criteria to dependence, after standardization of age. The duration of the first criteria to dependence did not differ significantly (0.49 years for alcohol versus 0.64 years for opioids) between both groups. Similarly the duration of the second criteria to the development of dependence also did not differ significantly (0.24 years for alcohol versus 0.28 years for opioids) (summarized in Table 2).

**Table 2:** Clinical course of dependence

Factors	Alcohol no. (%)/Mean ± S.D (N=81)	Opioids no. (%)/Mean ± S.D (N=31)	χ^2^/t-test	df	Significance *p*
Age at onset of substance use	18.72 ± 6.84	20.73 ± 3.93	1.937	110	0.05
Age at appearance of first criteria	24.33 ± 9.21	21.39 ± 3.94	1.710	110	0.09
Age at appearance of ICD 10 dependence	27.51 ± 9.28	22.05 ± 3.98	3.159	110	0.002*
Duration from onset to dependence	8.78 ± 6.7	1.32 ± 0.89	6.162	110	<0.001**
Duration from first criteria to dependence	0.49 ± 0.3	0.65 ± 0.4	1.925	43	0.061
Duration from second criteria to dependence	0.24 ± 0.3	0.28 ± 0.2	0.915	57	0.364

T-test has been used for continuous variables and Chi-square for categorical variables;

* significance at *P*<0.05

** Significance at *P*<0.001

## Discussion

Although the legal age of alcohol use in many states in India is 25 years,(27) most studies done earlier as well as ours has observed a downward trend in the ages of onset of both alcohol and opioid use which was earlier noted to be 20-25 years for alcohol(28–31) and 23 - 30 years for opioids.(32,33) Across South East Asia, the legal age at which alcohol may be served varies from a low of 18 years in Bhutan, Myanmar, Nepal, Sri Lanka and Thailand to 21 years in Indonesia and a high of 25 years in India,(34) although implementation of the law in India remains a major concern. Since there is evidence that simply raising the legal age limit may help in reducing alcohol-related problems and the consumption of alcohol by minors,(35) an uniform cut-off age across the whole region can significantly reduce the sale of alcoholic beverages to underage young people.(36)

This study also noted that there is a *rapid progress from onset to dependence* noted within just 8.78 years in alcohol-dependent subjects although both alcohol and opioid use start almost simultaneously (18 versus 20 years), calling for a serious introspection. There is also a similarity in the pattern of appearance of criteria, from first to second to dependence for both substances. Further, the three most common criteria appearing before development of dependence, namely, craving, tolerance, and withdrawal, are also similar for both groups (data not shown). Similar patterns of dependence along with the rapid downhill course seen in the alcohol group forces one to wonder if legal controls should not be in place to prevent progression of the pre-dependence stages to dependence.

Previous studies have observed excessive use of alcohol in these pre-dependence stages,(37–39) which would be defined in terms of exceeding a certain daily volume (e.g., three drinks a day) or quantity per occasion (e.g., five drinks on an occasion, at least once a week), or daily drinking.(40) Such persistent patterns of drinking may result in acute or chronic medical, psychiatric, and economic consequences(41) on the drinker in question. Research has also shown that when extrapolating from historical trends, the role of alcohol as a major factor in the burden of disease will be increasing in the future. This is also accompanied by worrying trends of increases in the average volume of drinking predicted for the most populous regions of the world (e.g., in China and India) and the emerging trend of more harmful and risky patterns in drinking, especially among young people.(42)

Yet the absence of legal control of alcohol is surprising, despite clear evidence that this has resulted in lower levels of consumption,(43) reduced drink-driving casualties and violence.(44,45) Unfortunately the legislation varies widely across the South East Asian countries, given the fact that patterns of use and dependence are similar.(46) For example, alcohol use is restricted on religious grounds in the predominantly Muslim countries of Bangladesh and Maldives. Yet, the almost constant consumption of alcohol in Bangladesh over the years indicates that alcohol is available to users across all strata of the population, albeit in small quantities.(34)In the other South East Asian countries, drunken driving is legally forbidden in all countries, although both Indonesia and Bhutan are yet to establish a maximum Blood Alcohol Concentration (BAC) limit while driving. The maximum legal BACs also show a wide variation ranging from 30-70 mg% in different countries. The law on public consumption of alcohol also varies with minimal restrictions in Bhutan and Myanmar, partial restriction in Sri Lanka ad Thailand and complete restriction in India. This wide disparity in legal controls calls for a uniform law on the lines of the Framework Convention for Tobacco Control,(47) passed and ratified by member countries of the WHO. However, since public legislation is often influenced by community attitudes and consensus in opinion, it may be worthwhile exploring avenues for legislation to control the hazardous (or pre-dependence stage) use of alcohol.

To summarize, we believe that we have presented convincing arguments that opioids and alcohol be treated similarly. First, the ages of onset are similar for both substances. Second, the patterns of dependence, from both onset and appearance of the first criteria to dependence are similar. And finally, the likelihood of development of dependence among users is also similar (16% for alcohol versus 23% for opioids).(48) We therefore feel that there is a strong justification for the health professions to step up their health advocacy, with respect to policies, to reduce the rates of alcohol problems. The crucial need, from a public health perspective, is to consider some legislations especially for the hazardous (or pre-dependence stage) use of alcohol, a recommendation suggested even by the WHO.(49)

## Conclusion

Considering the downward trend in ages of onset and comparatively rapid progression to dependence once the first and second criteria appear, and in spite of societal acceptance and arguments in favor of the cardio-protective effect of moderate doses of alcohol, we feel that legislations should be debated/considered, especially for the hazardous use of alcohol, with the enactment of a uniform law across the world. An international consensus has to be evolved on alcohol control policy and prohibition, and certain measures have to be advocated in line with the recommendations of the WHO.(49)

## References

[1] Crawford A, Hinton JW, Docherty CJ, Dishman DJ, Mulligan PE (1982). Alcohol and crime.I: Self-reported alcohol consumption of Scottish prisoners. J Stud Alcohol.

[2] Kershaw C, Budd T, Kinshott G, Mattinson J, Mayhew M, Myhill A (2000). The 2000 British Crime Survey, England and Wales, Home Office Research, Development and Statistics Directorate.

[3] Tether P, Harrison L (1986). Alcohol related fires and drownings. Br J Addict.

[4] Road Casualties Great Britain 2001- Department of Transport, Great Britain. The Stationery Office.

[5] World Health Report 2002: reducing risks, promoting healthy life http://whqlibdoc.who.int/hq/2002/WHO_WHR_02.1.pdf.

[6] Park K (2005). Mental Health.

[7] Anonymous Globalization and increasing trend of alcoholism.

[8] Saxena S (1997). http://www.who.int/substance_abuse/publications/en/india.pdf.

[9] (1997). Summary and recommendations. National Workshop on Alcohol Policy: Health Perspectives.

[10] Chagas Silva M, Gaunekar G, Patel V, Kukalekar DS, Fernandes J (2003). The prevalence and correlates of hazardous drinking in industrial workers: A study from Goa, India. Alcohol Alcohol.

[11] Gururaj G (2002). Epidemiology of traumatic brain injuries: Indian scenario. Neurol Res.

[12] Sri EV, Raguram R, Srivastava M (1997). Alcohol problems in a general hospital: A prevalence study. J Indian Med Assoc.

[13] Gururaj G, Girish N, Benegal V (2006). Burden and socio-economic impact of alcohol use: The Bangalore study. World Health Organization, Regional Office for South-East Asia.

[14] (1985). Narcotic Drugs and Psychotropic Substances Act (As amended upto date), Ministry of Law and Justice, Government of India.

[15] (1993). International Classification of Diseases 10th revision Diagnostic Criteria for Research.

[16] Folstein MF, Folstein SE, McHugh PR (1975). Mini-mental state-a practical method for grading the cognitive state of patients for the clinician. J Psychiatr Res.

[17] Bucholz KK, Cadoret R, Cloninger CR, Dinwiddie SH, Hesselbrock VM, Nurnberger JI (1994). A new, semi-structured psychiatric interview for the use in genetic linkage studies: a report on the reliability of the SSAGA. J Stud Alcohol.

[18] Hesselbrock M, Easton C, Bucholz KK, Schuckit MA, Hesselbrock V (1999). A validity study of the SSAGA: A comparison with the SCAN. Addiction.

[19] Ehlers CL, Wall TL, Betancourt M, Gilder DA (2004). The clinical course of alcoholism in 243 Mission Indians. Am J Psychiatry.

[20] Schuckit MA, Anthenelli RM, Bucholz KK, Hesselbrock VM, Tipp J (1995). The time course of development of alcohol-related problems in men and women. J Stud Alcohol.

[21] Hesselbrock VM, Segal B, Hesselbrock MN (2000). Alcohol dependence among Alaska Natives entering alcoholism treatment: A gender comparison. J Stud Alcohol.

[22] Wall TL, Carr LG, Ehlers CL (2003). Protective association of genetic variation in alcohol dehydrogenase with alcohol dependence in Native American Mission Indians. Am J Psychiatry.

[23] Nelson EC, Heath AC, Bucholz KK, Madden PA, Fu Q, Knopik V (2004). Genetic epidemiology of alcohol-induced blackouts. Arch Gen Psychiatry.

[24] Culverhouse R, Bucholz KK, Crowe RR, Hesselbrock V, Nurnberger JI, Porjesz B (2005). Long-term stability of alcohol and other substance dependence diagnoses and habitual smoking: An evaluation after 5 years. Arch Gen Psychiatry.

[25] Wing JK, Babor T, Brugha T, Burke J, Cooper JE, Giel R (1990). SCAN-schedules for clinical assessment in neuropsychiatry. Arch Gen Psychiatry.

[26] Carlat DJ (2005). Asking questions III: Tricks for improving patient recall. The psychiatric interview: A practical guide.

[27] Global Status Report on Alcohol http://www.who.int/entity/substance_abuse/publications/global_status_report_2004_overview.pdf.

[28] Singh J, Singh G, Mohan V, Padda AS (2000). A comparative study of prevalence of regular alcohol users among the male individuals in an urban and rural area of District Amritsar, Punjab. Indian J Community Med.

[29] Chaudhury, Das SK, Mishra BS, Ukil B, Bharadwaj P, Dinker NL (2002). Physiological assessment of male alcoholism. Indian J Psychiatry.

[30] Meena, Khanna P, Vohra AK, Rajput R (2002). Prevalence and pattern of alcohol and substance abuse in urban areas of Rohtak city. Indian J Psychiatry.

[31] Mattoo SK, Basu D (1997). Clinical Course of Alcohol dependence. Indian J Psychiatry.

[32] Mahanta J, Chaturvedi HK, Phukan RK (1997). Opium addiction in Assam: A trend analysis. Indian J Psychiatry.

[33] De B, Mattoo SK, Basu D (2002). Age at onset typology in opioid dependent men: An exploratory study. Indian J Psychiatry.

[34] (2006). Alcohol Control Policies in the South-East Asia Region: Selected Issues. WHO SEARO.

[35] Grube JW, Nygaard P (2001). Adolescent drinking and alcohol policy. Contemp Drug Prob.

[36] Wagenaar AC, Murray DM, Toomey TL (2000). Communities mobilizing for change on alcohol (CMCA): Effects of a randomized trial on arrests and traffic crashes. Addiction.

[37] Sarr M, Bucholz KK, Phelps DL (2000). Using cluster analysis of alcohol use disorders to investigate ‘diagnostic orphans’: Subjects with alcohol dependence symptoms but no diagnosis. Drug Alcohol Depend.

[38] Pollock NK, Martin CS (1999). Diagnostic orphans: Adolescent with alcohol symptoms who do not qualify for DSMIV abuse or dependence diagnoses. Am J Psychiatry.

[39] Eng MY, Schuckit MA, Smith TL (2003). A five-year prospective study of diagnostic orphans for alcohol use disorders. J Stud Alcohol.

[40] Babor TF, Caetano R, Casswell S, Edwards G, Giesbrecht N, Graham K (2003). Alcohol: No Ordinary Commodity-Research and Public Policy.

[41] Room R, Babor T, Rehm J (2005). Alcohol and public health. Lancet.

[42] (2004). World Health Organization Global Status Report on Alcohol. Department of Mental Health and Substance Abuse.

[43] Mäkelä K, Room R, Single E, Sulkunen P, Walsh B (1981). Alcohol, society and the state: Vol. 1. A comparative study of alcohol control.

[44] Holder HD, Wagenaar AC (2004). Mandated server training and reduced alcohol-involved traffic crashes: A time series analysis of the Oregon experience. Accid Anal Prev.

[45] Wallin E, Norström T, Andréasson S (2003). Alcohol prevention targeting licensed premises: A study of effects on violence. J Stud Alcohol.

[46] Alcohol, gender and drinking problems: A perspective from low and middle income countries, Department of Mental Health and Substance Abuse.

[47] (2005). World Health Organization: Framework convention on tobacco control.

[48] Jaffe HJ, Anthony CJ, Sadock BJ, Sadock VA (2005). Substance related disorders: Introduction and Overview. Kaplan and Sadock's Comprehensive Textbook of Psychiatry.

[49] Alcohol consumption control - policy options in the South-East Asia region.

